# Preparation of Environmentally Friendly Oil- and Water-Resistant Paper Using Holo-Lignocellulosic Nanofibril (LCNF)-Based Composite Coating

**DOI:** 10.3390/polym16081078

**Published:** 2024-04-12

**Authors:** Shengdan Wang, Lihua Pei, Jichao Wei, Jiabao Xie, Xingxiang Ji, Yukang Wang, Peng Jia, Yajuan Jiao

**Affiliations:** 1 State Key Laboratory of Biobased Material and Green Papermaking, Key Laboratory of Pulp and Paper Science & Technology of Ministry of Education/Shandong Province, Qilu University of Technology (Shandong Academy of Sciences), Jinan 250353, China; wsd6849@126.com (S.W.);; 2 Dongying Huatai Chemical Industry Group Co., Ltd., Dongying 257000, China; 3 Shandong Dingan Testing Co., Ltd., Jinan 250353, China; 4 Shandong Textile & Architecture Design Institute Co., Ltd., Jinan 250353, China

**Keywords:** holo-lignocellulosic nanofibrils, oil-resistant, water-resistant, coating, packaging paper

## Abstract

In the present study, an environmentally friendly oil- and water-resistant paper was developed using a holo-lignocellulosic nanofibril (LCNF)-based composite coating. The LCNF was prepared from wheat straw using a biomechanical method. Characterizations of oil- and water-resistant coated paper and the effect of LCNF content on the performance of the coated paper were confirmed by combining contact angle analysis, Cobb 300s, and mechanical performance tests. The results show that the barrier performance and mechanical strength of the coated paper were greatly improved with the increase of LCNF content. The contact angle of oil and water of coated paper containing 50% LCNF were 69° and 78°, respectively, while the contact angle of oil and water of the base paper were only 30° and 20°, respectively. Cobb 300s values reduced from 110 g/m^2^ to 30 g/m^2^ when the LCNF content increased from 50% to 90%. Moreover, under the coating amount of 20 g/m^2^, the tensile strength of the coating paper was 0.980 KN/m, an increase of 10.11% compared with the base paper. The bursting strength reached 701.930 KPa, which was 10.75% higher than the base paper. In short, it is feasible to prepare LCNF from wheat straw, and apply it to produce water-proof and oil-proof paper. The water-proof and oil-proof paper developed in this study not only offers a novel approach to addressing white pollution but also presents a new research avenue for exploring the potential applications of agricultural waste.

## 1. Introduction

Disposable plastic products, renowned for their affordability, corrosion resistance, high strength, and water-resistant properties, are commonly utilized in daily life. However, plastic products are difficult to biodegrade in nature, which seriously endangers the ecological environment and human health. In response to this issue, over 40 nations worldwide have implemented plastic bans and stricter regulations on plastic products [1]. Therefore, the development of renewable products to replace single-use plastic packaging products have become a hot topic widely studied by scientists [2]. At the same time, research on paper-based materials instead of disposable plastic products has become a new research direction in the field of biodegradable products [3,4]. However, the hydrophilic and oil-friendly characteristics of paper limit its direct use as a substitute for disposable plastic products. Paper’s water resistance and oil resistance are primarily enhanced by adding functional fillers in the papermaking process, directly modifying the paper, and applying functional coatings on its surface. Of these methods, the method of coating the surface of paper is the most practical for actual production due to its simplicity, low cost, and high flexibility [5]. At present, most of the surfaces of water-resistant and oil-resistant paper materials contain plastic film or silicon/fluoropolymer coatings, which are still seriously harmful to the environment. Therefore, the development of environmentally friendly and efficient water-resistant and oil-resistant paper-based coatings is the key to replacing disposable plastics.

Cellulose nanofibrils (CNF) have been widely studied for their potential as substitutes for synthetic and non-biodegradable polymers in various applications. In most studies on CNF, the expensive bleached cellulosic pulps are commonly used as the raw resource. However, compared to the production of CNF from bleached pulp, holo-lignocellulosic nanofibrils (LCNF) prepared using unbleached raw resources contain residual lignin, and exhibit better hydrophobic and thermal stability. The presence of residual lignin in the nanofibril matrix can confer unique polar, hydrophobic, and thermal stability to LCNF, making it compatible with various hydrophobic polymers. Furthermore, using unbleached raw resources as the raw material for the preparation of LCNF can improve the yield and reduce the costs and environmental impact [6]. Thus, the development of LCNF-based water-resistant and oil-resistant coating should be thoroughly studied. Although wood is the typical raw material used for LCNF preparation, excessive logging can damage the ecological environment. Therefore, grass-based raw materials and other solid agricultural and forestry waste, as well as industrial cellulose-containing by-products with abundant reserves, represent potential alternative raw materials for the preparation of LCNF [2,7,8,9,10].

As an agricultural waste product, wheat straw has several advantages, including a wide range of sources, high yield, and low price [11]. Additionally, it has high holocellulose and lignin content, short and thin fibers, a loose and porous structure, and a relatively large wall cavity, making it a promising resource for biomass utilization [7]. However, the natural cuticle on the surface of wheat straw can hinder swelling and softening of the wheat straw fiber. To address this issue, an alkaline hot water pre-impregnation method is commonly used to dissolve some pectin, wax, and other substances in wheat straw, improving the accessibility of the drug liquid during the subsequent mechanical treatment process. Other pretreatment methods are also available for wheat straw pretreatment. For example, mechanical pretreatment can mechanically destroy the structure of wheat straw, enhancing the accessibility and swelling effect of fibers. Alternatively, biological pretreatment employs the action of microorganisms to decompose the wax on the surface of wheat straw, improving the swelling and softening effect of internal fibers [11]. By using appropriate pretreatment methods, high-quality LCNF can be extracted from wheat straw and used to prepare water-resistant and oil-resistant coatings. 

In our previous work, a LCNF-based composite multifunctional superhydrophobic coating was fabricated using an efficient silylation process from bleached wood pulp, tetraethyl orthosilicate, and cetyl trimethoxysilane [12]. However, the application of silicon-containing polymers in the field of food packaging has been controversial. To enhance the safety and environmental friendliness of paper-based water- and oil-resistant coatings, in this study, LCNF and water-resistant and oil-resistant coatings were prepared with a bio-mechanical method using wheat straw. Subsequently, environmentally friendly water-resistant and oil-resistant paper was developed using the LCNF-based coating, aiming to replace traditional plastic packaging.

## 2. Materials and Methods

### 2.1. Materials

Wheat straw (scrubbed) was taken from a paper mill in Weifang, Shandong Province, containing 61.8% holocellulose, 27.2% lignin, and 6.41 ash. The wheat straw was cut into 40–60 mm long sections as raw material. The base paper was supplied by a paper mill (Weifang, China). Commercial cellulase (Banzyme 2900, activity 7.3 IU/mL) was purchased from UPM-kymmene Co., Ltd. (Changshu, China). The optimum pH and temperature for the cellulase were 5.5 and 50 °C, respectively. Cationic starch was supplied by Shandong Huatai Paper Co., Ltd. (Dongying, China). All other chemicals used were obtained from Macklin Biochemical Co., Ltd. (Shanghai, China). 

### 2.2. Preparation of LCNF

A certain amount of wheat straw was suspended in 100 °C alkaline hot water (alkali content: 2%, liquid ratio of 1:4). The mixture of samples was incubated at 100 °C for 30 min. After alkaline hot water pre-impregnation, the straw was washed many times until the pH value of the filtrate approached 7.0. The above alkaline hot water pre-impregnation sample was ground in two stages with a high-consistency disc refiner (2500-II, KRK Corporation, Tokyo, Japan). The gap between the grinding pieces was 0.5 mm and 0.2 mm. 

After the mechanical treatment, the sample was washed several times. Enzymatic treatment of the sample was conducted according to the procedure in our previous work [11]. Briefly, 2% (*w*/*v*) pulp was dispersed in 50 mM of citrate acid–sodium citrate buffer (pH 4.8) with cellulase loading of 50 mg/g substrate. The slurry was incubated at 50 °C for 1.5 h. After that, the slurry was centrifuged to separate the solid and liquid phases. The solid phases were washed many times until the pH value of the filtrate approached 7.0. 

After enzymatic treatment, size reduction of the sample into microfiber/nanofiber cellulose was prepared according to the procedure in our previous work [13]. Briefly, the pulp at 1 wt% was mechanically treated by a supermass colloider (MKCA6-5 J, Masuko Sangyo Co., Ltd., Saitama, Japan). The obtained slurry was further diluted to 0.5 wt% and treated with a high-pressure Nano DeBEE slit homogenizer (BEE International, South Easton, MA, USA). LCNF was obtained after 15 passes through the homogenizer at the slit pressure of 25,000 psi.

### 2.3. Preparation of Oil- and Water-Resistant Paper

Cationic starch of 25 g and sodium hydroxide of 2.5 g were dissolved in 150 g and 22.5 g deionized water, respectively. The dissolved starch slurry was incubated in a 40 °C constant temperature water bath. Under continuous high-speed stirring (12,000 rpm) with an emulsifier, the sodium hydroxide solution was dropped into the starch slurry. After all the sodium hydroxide solution was added, it was stirred continually for 5 min. Then, the above starch slurry with certain amounts (50%, 60%, 70%, 80%, and 90%) of LCNF was mixed, and emulsified at 10,000 rpm for 5 min. After that, an LCNF-based composite coating was obtained. 

**Figure 1:**
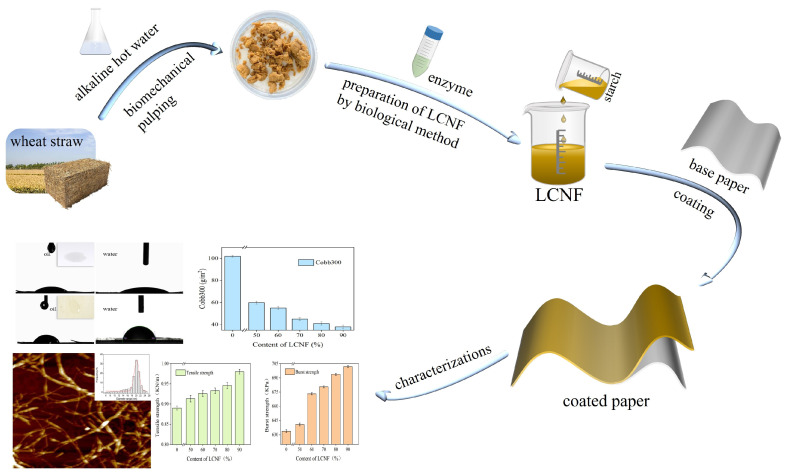
Production process flow chart of the oil- and water-resistant coated paper.

The base paper was coated with the LCNF-based composite coating using coating rods of different thicknesses (6 μm and 10 μm). The coating was carried out manually. The coated paper was dried at 100 °C in an oven to create oil- and water-resistant paper. Figure 1 presents a production process flow chart of the water-/oil-resistant paper.

### 2.4. Characterizations of the LCNF

Morphology: The particle size distribution of the LCNF was investigated by atomic force microscopy (AFM). A drop of diluted LCNF suspension (0.01 wt%) was allowed to dry on a clean mica substrate at room temperature for 24 h. AFM images were shown in the height mode without other image processing except flattening. The surface morphologies of the LCNF were observed using field emission scanning electron microscopy (FE-SEM, Hitachi Regulus 8220, Tokyo, Japan). A drop of diluted LCNF suspension (0.1 wt%) was oven-dried at 40 °C and coated with a thin layer of gold before the observation.

Crystalline structure: The crystalline structures of wheat straw and LCNF were determined by an X-ray diffractometer (XRD, D/max-IIIA, Rigaku, Tokyo, Japan), using Cu–Ka radiation at a scan rate of 2°/min. The samples were scanned at 40 kV and 30 mA in a 2θ range between 5° and 60°. The crystallinity index of wheat straw and LCNF were calculated as follows [14]:

(1) $$\mathrm{Cr}=\frac{I_{002}-I_{am}}{I_{002}}\times 100\%$$

where Cr is the crystallinity index; *I*_002_ is the diffraction peak intensity of the crystalline region at 2θ ≈ 22.6°; and *I*_am_ is the minimum diffraction intensity at 2θ ≈ 18.6°. 

Mechanical properties of the LCNF films: In order to prepare neat LCNF films, LCNF films of different quantifications (20, 40 and 60 g/m^2^) were produced from LCNF suspensions (0.2 wt%) using the vacuum filtration method over a 0.45 μm nitrocellulose membrane filter (CA, Shanghai, China). After filtration, the wet sheet was then drained at 60 °C under a pressure of approximately 25 kg for 3 h. Finally, the obtained films were stored at 23 °C and 50% RH for 24 h. The tensile properties of LCNF films were tested using a universal testing machine (Instron 3365, Instron, Canton, NY, USA) at 1 mm/min. The dimensions of the samples were 30 mm for the length and 5 mm for the width. Prior to mechanical testing, specimens were pre-conditioned at 25 °C and 50% relative humidity for 24 h.

Thermal stability: The thermal decomposition performance of LCNF was analyzed in a nitrogen atmosphere at a heating rate of 10 °C/min using a simultaneous thermal analyzer (STA446 F3).

### 2.5. Characterizations of Oil- and Water-Resistant Paper

Surface morphology: The surface microstructure and morphology of the base paper and coated paper were observed using a scanning electron microscope (SEM; Zeiss; EVO18, Tokyo, Japan). All samples were sprayed with a thin layer of gold to ensure conductivity before testing.

Contact angle analysis: The water contact angle and oil contact angle were measured using an LSA100 contact angle meter (LAUDA Scientific, Lauda-Königshofen, Germany) at ambient temperature. For these measurements, 5 μL of deionized water droplets (or oil droplets) were carefully placed on the substrate using a high-precision syringe, and kept for 5 s before measuring the contact angle. The oil was prepared according to the Kit (test) solutions using mixtures of castor oil, toluene, and n-heptane. Contact angles were repeated five times at different positions. 

Oil resistance test: The oil resistance was measured according to the standard Tappi T559 pm-96. The coated paper was tested with a series of solutions with different Kit numbers (1–12), which contained specific proportions of three reagents: castor oil, toluene, and n-heptane. Oil number 1 was the least aggressive oil, i.e., with the highest surface energy, and oil number 12 was the most aggressive oil, i.e., with the lowest surface energy. The solution was dropped onto the coated paper surface from a predetermined height. After 15 s, oils were removed with oil-absorbing sheet. The highest numbered liquid that remained on the surface of the paper sample, without causing staining, was reported as the Kit value for the coated paper.

Water resistance test: The Cobb value (Cobb 300s) was measured according to international standards (ISO 535: 2014, paper and board—determination of water absorptiveness—Cobb method) [15]. The samples were cut into a specimen with a diameter of (125 ± 5) mm. Then, 30 mL of water was added to the cylinder (the internal area was 100 cm^2^). The cut sample was placed on the annular surface of the cylinder, tightly covered, and then rotated 180° to make full contact the water in the cylinder. The sample was removed after 300 s. The water-absorbing surface of the sample was placed downward on the pre-laid absorbent paper. A piece of absorbent paper was placed on the sample, and a metal pressure roller (cylinder accessory) was immediately used to roll back and forth within 4 s without any other pressure. Then, the sample was quickly taken out, and the absorbent surface was folded inward, and then folded in half again and weighed. The Cobb value was repeated five times to ensure the accuracy of the experiment. The water uptake was determined according to the following equation:

(2) $$C=\left(m_{2}-m_{1}\right)\times 100$$

where *C* is the Cobb value (unit: g/m^2^), *m*_1_ is the initial weight of the sample before immersion; and *m*_2_ is the weight measured after immersing the sample in water for 300 s, and removing the excess water.

Mechanical performance analysis: The mechanical performance tests were carried out on the base papers and coated papers. The tensile strength and bursting strength of paper sheets were measured by a tensile strength tester (L066-5135, L&W Corporation, Kista, Sweden) and a bursting strength tester (BSM-1600B, L&W Corporation, Sweden), according to GB/T 12914-2018 [16], and GB/T 454-2020 [17] for determination. The sample for paper tensile strength testing was sampled along the longitudinal direction of the paper. The dimensions of the samples were 180 mm for the length and 15 mm for the width. The tensile properties of the base paper and coated paper were tested at 20 mm/min. The bursting strength tester is mainly composed of clamping, transmission, pressure, and indication systems. Due to the significant impact of clamping force on the test results, the clamping force should not be less than 430 kPa when testing the bursting strength of paper. The rubber film is round and made of rubber material. The upper surface of the rubber film is about 3.5 mm lower than the upper surface of the lower chuck when it is static. The elastic resistance of the rubber film is large, and when the rubber film protrudes from the lower chuck surface by (9 ± 0.2) mm, its pressure is (30 ± 5) kPa. The hydraulic system and the liquid used should be free of air bubbles, and the pumping volume should be (95 ± 5) mL/min. When the bursting strength tester is static, the upper surface of the rubber film is about 3.5 mm lower than the upper surface of the lower chuck. The elastic resistance of the rubber film is large, and when the rubber film protrudes from the lower chuck surface by (9 ± 0.2) mm, its pressure is (30 ± 5) kPa. When testing the bursting strength of paper, the sample should be placed between the upper and lower clamping rings of the calibrated bursting strength tester, and clamped. The control knob should be dialed to move the piston, maintaining an oil inlet speed of 95 mL/min. The strength test for each sample was performed 10 times, and the results were averaged. Prior to mechanical testing, specimens were pre-conditioned at 25 °C and 50% relative humidity for 24 h.

## 3. Results

### 3.1. Properties of the LCNF

**Figure 2:**
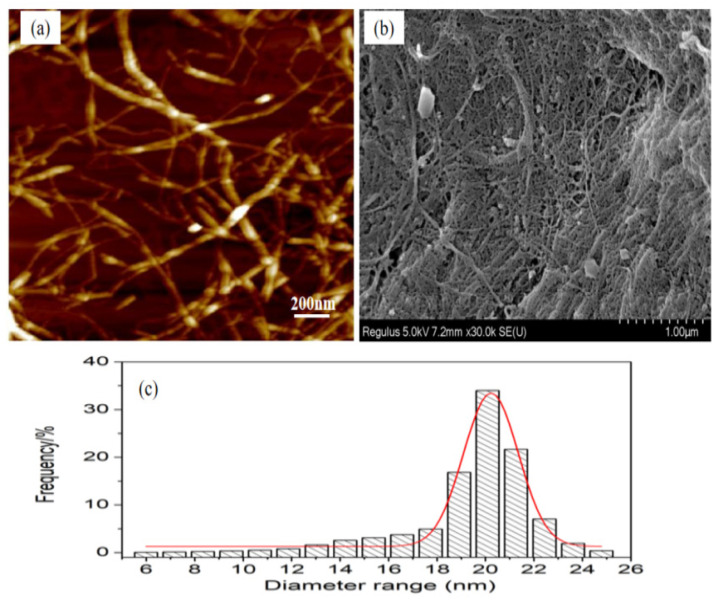
(**a**) AFM image of LCNF; (**b**) SEM image of LCNF; (**c**) particle size of LCNF.

The particle size of the LCNF was shown in an AFM image (Figure 2a). The diameter distribution of the LCNF in the AFM image was statistically analyzed to be within the range of 16–26 nm (Figure 2c), using a nanoscope analysis software (version 1.2), while the length was up to several microns. Therefore, the sample could be referred to as nanofibers [18]. The surface morphology of the LCNF was shown in a SEM image (Figure 2b). The web-like network and agglomeration structure of the LCNF can be seen in the SEM image. In addition, some lignin particles were observed [19].

**Figure 3:**
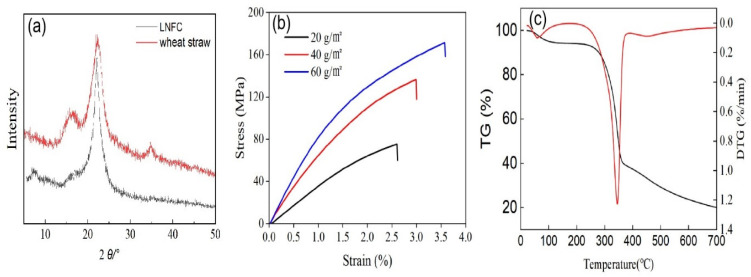
The XRD spectra of LCNF and wheat straw (**a**); tensile strength of LCNF films (**b**); thermogravimetric and derivative curves of LCNF (**c**).

The crystallinity of wheat straw would change with alkaline hot water pre-impregnation, mechanical treatment, and enzyme treatment due to the higher crystallinities enhancing the mechanical properties of nanocomposites [20]. The crystallinities of wheat straw and LCNF were measured. Figure 3a shows that the crystallinity of LCNF was 51.80%, which was higher than the 48.35% crystallinity of wheat straw. This was because hot water pre-impregnation and enzyme treatment removed some non-crystalline parts of the wheat straw substrate. However, compared with wheat straw, the crystallinity of LCNF only increased by 3.45. This occurred because mechanical treatment (high-pressure homogenization) can destroy the ordered crystalline zone structure of cellulose by strong shear force, and reduce the crystallinity [21,22].

The tensile strength and strain curves of LCNF films for different quantifications (20, 40 and 60 g/m^2^) are shown in Figure 3b. The high quantification (60 g/m^2^) LCNF film presented a higher tensile strength (171.5 MPa) and strain (3.57%), which increased by 93.2 MPa in comparison with the low quantification (20 g/m^2^). The tensile strength of LCNF film prepared from wheat straw showed excellent tensile strength [23,24].

To analyze the thermal stability of LCNF, the initial pyrolysis temperature (T_onset_) and maximum pyrolysis temperature (T_max_) of LCNF were measured by thermogravimetric analysis. As can be seen from Figure 3c, the TG and DTG curves showed the typical trends of lignocellulosic materials. The thermal degradation of LCNF can be roughly divided into three stages: In the first stage, there was slight weight loss at 100 °C, primarily attributed to the evaporation of water absorbed by the sample; in the second stage, there was slow weight change between 100 °C and 265 °C, due to the lignin in LCNF containing phenolic hydroxyl and carbonyl groups being difficult to pyrolyze, which improved the thermal stability of LCNF; there was slow weight change between 100 °C and 265 °C, because lignin in LCNF containing phenolic hydroxyl and carbonyl groups were difficult to pyrolyze, which improved the thermal stability of LCNF; at the temperature of 265~500 °C in the third stage, the sample weight decreased greatly, which was caused by the rupture of carbon–carbon double bonds and the branched chains connected by aromatic groups in lignin [25,26]. Jonoobi et al. summarized that the T_onset_ and the T_max_ of LCNF prepared with different raw materials and methods ranged from 209 °C to 335 °C and 300 °C to 351 °C, respectively. In this study, the T_onset_ and T_max_ of the LCNF from wheat straw were 265 °C and 336 °C, respectively. The thermal stability of the LCNF prepared in this study was consistent with the results reported in the literature [27].

### 3.2. Surface Morphology of Paper

**Figure 4:**
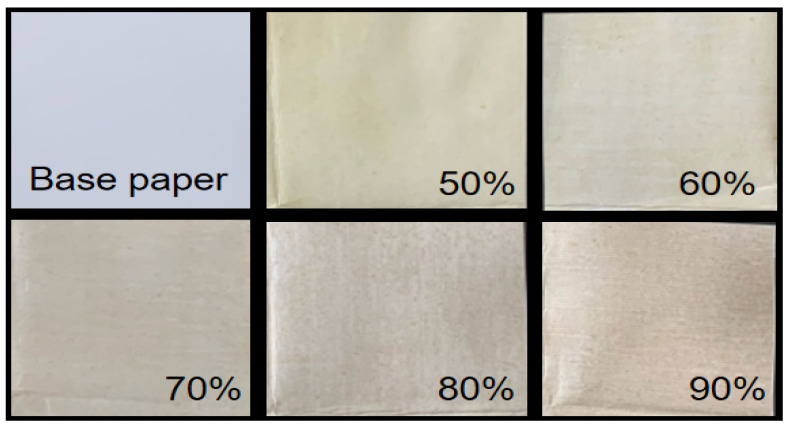
Photos of base paper and coated paper, which were coated with LCNF-based composite coating, containing different LCNF contents (50%, 60%, 70%, 80%, and 90%).

The unbeautified photos of the base paper and coated paper coatings with different LCNF contents, taken with Canon cameras, are shown in Figure 4. In the picture, the LCNF-based composite coating prepared by this study could be homogeneously distributed on the surface of the base paper. Furthermore, the color of the coated paper became deeper and darker with the increased LCNF content. Therefore, the applicable field of this coated paper would be limited to some extent. 

**Figure 5:**
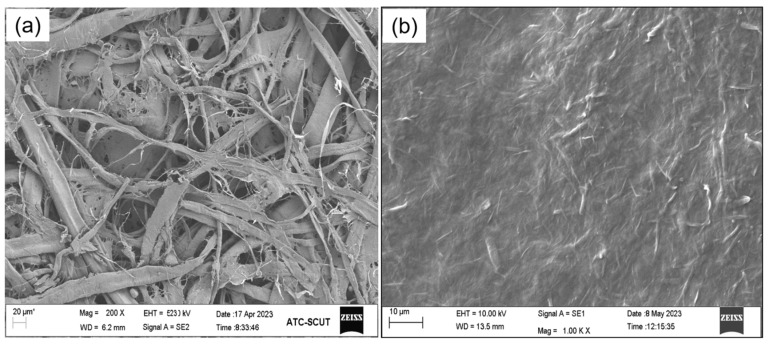
(**a**) SEM images of base paper; (**b**) coated paper with the LCNF-based composite coating (content 50% LCNF) under the coating amount of 20 g/m^2^.

The surface microstructure and coating distribution of the paper were further analyzed using SEM. The base paper and coated paper with the LCNF-based composite coating (50% LCNF content) under the coating amount of 20 g/m^2^ are shown in Figure 5a and b, respectively. The rough and porous structure of the base paper was apparently observed (Figure 5a). Compared to the base paper, the porous structure of the coated paper disappeared completely, and the surface of the paper became smooth. Similar results have been reported, that nanocellulose initially fills the large pores of the paper, and gradually forms a uniform and dense film on the surface of the paper as the nanocellulose load increases [27,28]. Therefore, CNF should be good for improving the gas barrier properties of papers [29].

### 3.3. Oil and Water Resistance of Coated Paper

**Figure 6:**
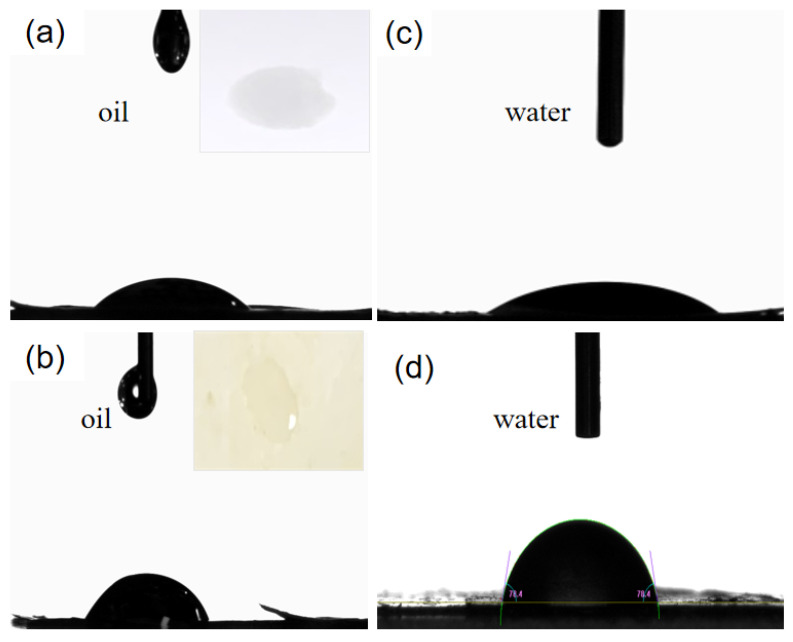
(**a**) The oil contact angle on the surface of base paper; (**b**) coated paper containing 50% LCNF; (**c**) the water contact angle on the surface of base paper; and (**d**) coated paper containing 50% LCNF.

The presence of residual lignin in the LCNF could confer unique polarity and hydrophobicity of LCNF-based composite coating, increasing its compatibility with cationic starch polymer. Therefore, the oil resistance and water resistance of coated paper with the lowest LCNF content of 50% were measured, according to the “barrel principle”. As shown in the upper right corner of Figure 6a, when an oil droplet was placed on the surface of the base paper, the oil easily penetrated the other side of the paper. Surprisingly, when the oil drops were placed on the surface of the coated paper, there was no penetration. At this time, the Kit value was 7, indicating that the oil protection grade of the coated paper was 7. Furthermore, the oil contact angle of the base paper and coated paper containing 50% LCNF were 30°and 69°, respectively, as shown in Figure 6a (base paper) and b (coated paper). It can be seen that the LCNF-based composite coating prepared in this study containing 50% LCNF had a better oil resistance effect. 

**Figure 7:**
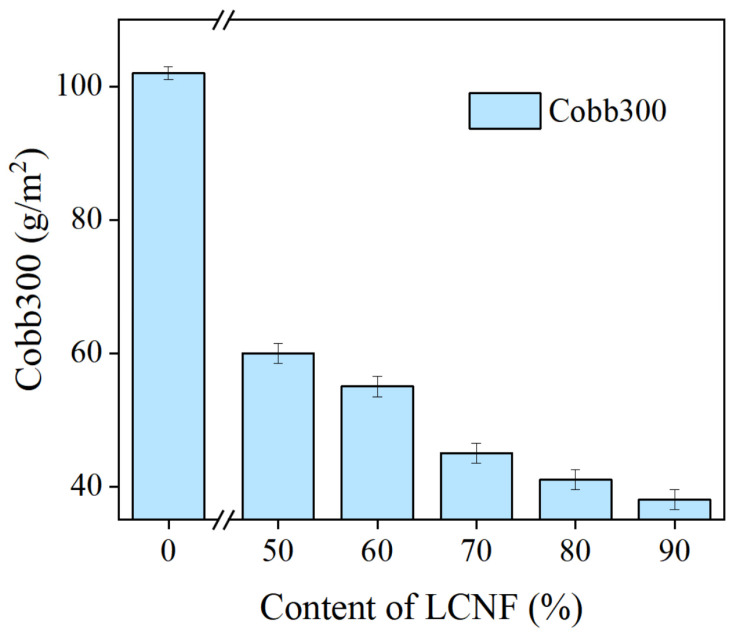
Water resistance of coated paper.

The water contact angle of base paper and coated paper containing 50% LCNF were 20° and 78°, respectively, as shown in Figure 6c (base paper) and d (coated paper). Moreover, the water proofing properties of coated paper containing different LCNF contents (50%, 60%, 70%, 80%, and 90%) were characterized by the Cobb 300s value, as shown in Figure 7. Cobb 300s values reduced from 110 g/m^2^ to 30 g/m^2^ when the LCNF content increased from 50% to 90%. The results show that a lower LCNF content of 50% contributed to the water resistance of the paper, which considerably improved at a higher LCNF content of 90%. The barrier performance of base paper was greatly improved with the increase of LCNF content, which may be due to the presence of hydrogen bonds in LCNF, meaning that a dense membrane structure can be formed between the fibers; or that more non-absorbable holes were produced in LCNF due to the presence of lignin particles. In addition, the LCNF fiber produced by the biomechanical method was more refined, and the bond between the fibers increased, resulting in an increase in density of the coating [30].

### 3.4. Mechanical Property of Coated Paper

**Figure 8:**
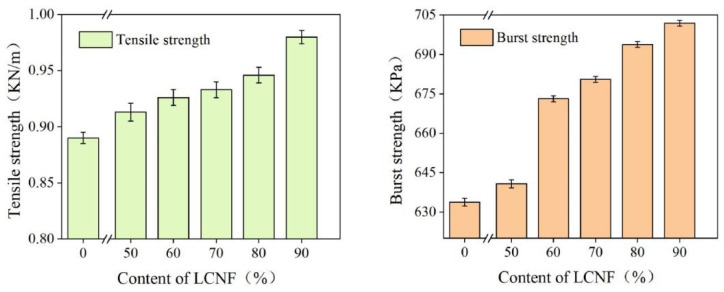
The tensile strength and bursting strength of base paper and coated paper.

The results of the analysis of the tensile strength and bursting strength of base paper and coated paper are shown in Figure 8. The tensile strength of base paper was 0.89 KN/m. Under the coating amount of 20 g/m^2^, with the increase of LCNF content in the coating, the tensile strength of the coated paper also increased. When the LCNF content in the coating was 90%, the tensile strength of the coating paper was 0.98 KN/m, an increase of 10.11% compared with base paper. Similarly, the bursting strength of base paper was 633.76 KPa. Under the coating amount of 20 g/m^2^, the bursting strength of coated paper gradually increased with the increase of LCNF content. When the LCNF content in the coating was 90%, the bursting strength reached 701.93 KPa, which was 10.75% higher than base paper. The mechanical property of paper mainly depends on the binding force between the fibers and the properties of fibers [31]. The improvement of the physical strength of coated paper might be due to enzyme pretreatment increasing the crystallinity of the LCNF and improving the bond between nanofibrils; or due to the wheat straw pulp being constantly divided into a broom during the mechanical grinding process, and more hydrogen bonds being exposed, so that fine fibers were connected.

## 4. Conclusions

Using a biomechanical method, holo-lignocellulosic nanofibrils (LCNF) were successfully produced from wheat straw. These LCNF had an average diameter of 14–16 nm and a high lignin content. The LCNF exhibited excellent hydrophobicity and mechanical properties owing to the presence of lignin. By combining LCNF with gelatinized starch, an LCNF-based composite coating was obtained, which can be used for paper coating. Base paper coated with the LCNF-based composite coating not only exhibited excellent barrier properties against water and oil, but also had significantly improved mechanical strength. The contact angles of oil and water of coated paper containing 50% LCNF were 69° and 78°, respectively, while the contact angles of oil and water of base paper were only 30° and 20°, respectively. Cobb 300s values reduced from 110 g/m^2^ to 30 g/m^2^ when the LCNF content increased from 50% to 90%. Moreover, under the coating amount of 20 g/m^2^, the tensile strength of the coating paper was 0.98 KN/m, an increase of 10.11% compared with base paper. The bursting strength reached 701.93 KPa, which was 10.75% higher than base paper. It is feasible to prepare LCNF from wheat straw, and apply these LCNF to create water-proof and oil-proof paper, although the color of the coated paper becomes deeper and darker as the LCNF content increases. Future research on LCNF-based composite materials will provide valuable theoretical support for the development of paper as an alternative to single-use plastic packaging products, and will also contribute to the high-value utilization of agricultural waste.

## Data Availability

The data are publicly available due to all data are contained within the article.
